# Problematic Reliance on Generative AI in an Anxious Young Adult: Case Report

**DOI:** 10.2196/98297

**Published:** 2026-09-16

**Authors:** Sarah Armstrong

**Affiliations:** 1 Department of Psychiatry, Schulich School of Medicine and Dentistry, Western University, 54 Riverview Ave, London, ON, N6J1A2, Canada, 1 519-646-6000

**Keywords:** AI, large language models, ChatGPT, anxiety disorders, young adult, case report

## Abstract

Generative AI tools are increasingly being integrated into daily life, offering cognitive support across domains such as writing, decision-making, and social interpretation. While beneficial, excessive reliance on such tools may contribute to diminished confidence in one’s own thinking.

This report describes a woman in her mid-20s with generalized anxiety disorder and major depressive disorder who developed a pattern of functional dependence on generative AI tools—specifically ChatGPT (OpenAI). She historically had strong social, academic, and occupational functioning. Psychiatric consultation did not suggest the presence of a personality disorder, and although she was a high-achieving individual, clinically significant perfectionistic traits were not evident. Despite this, she increasingly relied on AI for routine cognitive and interpersonal tasks, including composing emails, interpreting social interactions, predicting the future, and making decisions, and she felt increasingly uncomfortable completing such tasks independently. This case highlights the potential danger of generative AI tools in reassurance-seeking behavior and how their use might compound anxiety. These behaviors were observed across both professional and personal contexts, suggesting that the patient’s AI use was not limited to task-specific assistance but reflected a generalized strategy for managing uncertainty and self-doubt. In this case, this behavioral pattern was characterized by cognitive offloading, reduced confidence in independent judgment, and reinforcement of externalized thinking processes, which led to the subjective belief of declining skill and compromised functional autonomy. The Interaction of Person-Affect-Cognition-Execution (I-PACE) model was used to describe this pattern of behavior. While repeatedly turning to a family member or friend for validation and reassurance might lead to interpersonal fatigue, using AI tools for this purpose has no such limits, and the tools are infinitely accessible. This might ultimately worsen a patient’s ability to tolerate uncertainty. Given the ubiquity of AI tools and the prevalence of anxiety disorders, clinicians may need to be aware of the role AI tools could potentially play in reassurance seeking and thus the perpetuation of anxiety.

## Introduction

Many individuals now turn to generative AI for assistance with everyday tasks, from drafting emails to interpreting text messages to making decisions. While AI tools may enhance productivity, concerns are emerging regarding the potential effects that overreliance on them may have on independent cognitive skills, particularly in populations that are already vulnerable to anxiety and self-doubt [1-3]. Reassurance seeking and compulsive checking are well-recognized features of anxiety disorders and are known to provide short-term relief while reinforcing long-term distress [4-8]. Traditionally, these behaviors occur in interpersonal contexts, such as seeking validation from friends, family members, or clinicians. The emergence of generative AI introduces a novel, always-available source of reassurance that may alter this dynamic.

While it has been argued that the problematic use of large language models does not fit neatly into current addiction models [9], there is concern about their potential impact on long-term mental health and well-being [10,11]. Here, the term *functional dependence* is used descriptively rather than diagnostically to describe a pattern of repeated use of generative AI that exceeds efficacy and convenience. This construct overlaps with but is conceptually different from cognitive offloading—which does not describe anxiety and the need for reassurance. In the case described here, functional dependence included reassurance seeking but also extended to the externalization of judgment.

**Figure 1.**
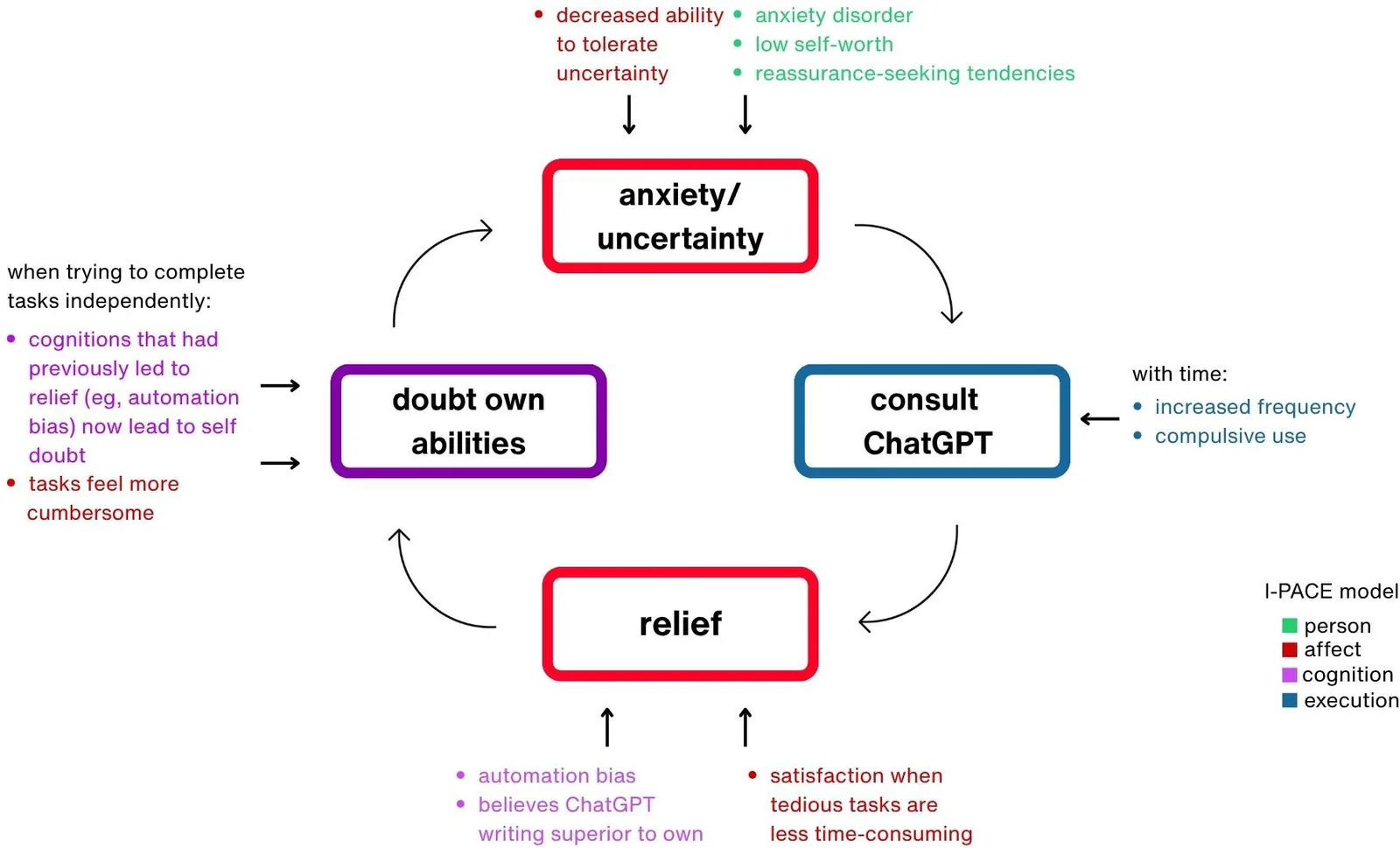
Reinforcement loop associated with ChatGPT use is illustrated, incorporating elements of Interaction of Person-Affect-Cognition-Execution (I-PACE) model.

The aim of this case report is to describe a pattern of problematic reliance on ChatGPT (OpenAI) in a young adult with anxiety. The Interaction of Person-Affect-Cognition-Execution (I-PACE) model is used in this case report to help conceptualize problematic use. The I-PACE model was originally proposed to explain internet-use disorders through the interplay of 4 core domains: personal predispositions, affective responses, cognitive processes, and behavior [12]. Although this case report does not argue for the diagnosis of an internet-use disorder, it does involve the problematic use of internet-based technology and contains observations corresponding to the 4 I-PACE domains (see Figure 1). Predisposing factors (person) are described. The negative thoughts and emotions (cognition/affect) are presented as potentially perpetuating factors that shape behavior (execution). By highlighting this emerging presentation, this report aims to increase awareness among mental health clinicians and researchers of a potential pattern of AI use that may warrant assessment in patients with anxiety disorders.

## Case Presentation

### Ethical Considerations

Written informed consent was obtained from the patient for publication of this case report and any accompanying details. Although the first name was changed for this report, all other details remain the same.

### Patient Profile and Presenting Concern

“Hailey” (a pseudonym) is a young woman in her mid-20s diagnosed with generalized anxiety disorder and major depressive disorder. Her symptoms are relatively well managed with medication and individual therapy, and she has never required psychiatric hospitalization. Although she struggles with pervasive low self-worth, her symptoms have never had a significant impact on her social, occupational, or academic functioning. She effectively managed a full university course load, and after graduating, she maintained employment while applying to graduate school. Historically, she sustained meaningful friendships and socialized often, and she continued to do so after treatment. Hailey often struggled with self-doubt, had a long-standing tendency to devalue her own opinion and judgment, and would often seek reassurance from others.

Hailey did not initially seek treatment for AI-related concerns. At first, her ChatGPT use did not appear problematic, but over time she became concerned about her frequency of use and the challenges she experienced in completing tasks independently. During one therapy appointment, she stated without prompting, “I’m worried I’m becoming dependent on ChatGPT.”

### Pattern of AI Use

Hailey completed high school and university without the use of AI. She indicated that writing essays was a task she excelled at. She was introduced to AI at work in the summer of 2024, when her boss recommended that she and her coworkers use the tool to write emails to customers to ensure that the tone was professional and generally sounded consistent. As illustrated in Figure 1, over the subsequent 2 years, she found herself increasingly turning to AI when feeling uncertain. She relied on ChatGPT rather than other generative AI models. Her use would vary depending on life circumstances. When faced with uncertainty (eg, applying to graduate school programs or going through a breakup), she would use it multiple times per day. During times when there was less uncertainty, she could go days without use.

### Written Communication

Hailey reported that she used AI to write professional emails approximately 90% of the time, which she would then edit herself. She wondered aloud during a therapy session about how she had ever been able to write email before AI. Her email-drafting process often involved multiple refinement cycles and hyperspecific tone calibration, with Hailey instructing ChatGPT not to make her sound “underachieving” but also not “over the top.” She often asked ChatGPT for reassurance about how she would be perceived and if the message was conveying too much of an undesired emotion (eg, “Does this read as aggressive?”). She would suggest multiple iterations, asking, “How about now?” When applying to graduate schools, she would ask ChatGPT what various programs were looking for in personal statements and seek reassurance that her personal statement was in line with these expectations.

Hailey would use ChatGPT to help craft personal text messages as well. Similarly, this would be triggered by anxiety about how she was being perceived. For example, she would upload a message from a romantic partner and ask how to respond. If uncertain about her partner’s feelings, she would ask ChatGPT to help create messages so she would not “spook” her romantic interest, with prompts such as “What’s a way to respond that seems natural, not too eager?”

### Social Interpretation

Hailey often used ChatGPT for social interpretation. If feeling uncertain in romantic relationships, Hailey would turn to ChatGPT, asking questions like, “What does he mean by this?” When dating someone new, she uploaded screen captures of a text conversation and asked ChatGPT if the new partner was losing interest. At times, she ran multiple different scenarios through AI.

Although less common, she would at times seek clarity about her own emotional responses. For example, she asked why a particular song was making her cry.

### Outcome Prediction

Hailey had difficulty tolerating uncertainty and would often turn to ChatGPT rather than wait for an outcome to occur. When applying to graduate schools, she would ask ChatGPT to make predictions about her likelihood of being accepted. She would ask questions such as, “Based on previous admissions cycles, should I have heard by now?” She would also ask the tool to predict what grade she would receive on various admission assessments.

When experiencing interpersonal conflict with friends or coworkers, she would enter the conversation into ChatGPT and ask it to predict the outcome. When coping with a breakup, she asked ChatGPT if she and her ex-boyfriend were going to get back together.

### Decision-Making

ChatGPT was also used to reduce uncertainty around decision-making. For larger life decisions, she would ask the tool to create a pro-and-con list. When applying for a new job, she would ask how to respond to questions about salary expectations. She also used ChatGPT for minor decisions that involved uncertainty. For example, after being invited to a formal event, she uploaded the invitation and asked what people would typically wear to such an event.

### Reading Comprehension of Professional Texts

Hailey found ChatGPT to be very helpful in breaking long text into more digestible pieces. Unlike the previous examples, this was typically motivated by wanting to save time rather than being driven by feelings of anxiety and uncertainty.

Notably, Hailey did not rely on ChatGPT across all domains. She did not use AI for tasks involving creativity or personal expression, such as planning a birthday celebration or selecting gifts, where she reported greater confidence in her own judgment.

### Impact of Use

Hailey began to view ChatGPT more as an authority than an assistant. Her use exceeded normal adaptation to the tool. She became distressed by her own use and described negative cognitions and decreased confidence in her own judgment and skills. In professional communication, she assumed that polished ChatGPT writing was superior to her own. She also assumed ChatGPT was an unbiased authority on how she would be perceived by others. From an affective perspective, she described feeling more confident in the short term when using ChatGPT. However, in the long term, she reported decreased confidence in her own independent judgment and abilities. Her description of this reinforced progression is illustrated in Figure 1. With time, her behavior, corresponding to the execution component of the I-PACE model, seemed less like a choice. She found herself reaching for ChatGPT without thinking about it. She would compulsively use it even when she knew it was not helping her. For example, asking the tool to predict the outcome of graduate school applications increased her distress, yet she had a hard time abstaining.

Hailey described using ChatGPT as an alternative to talking with friends, family, or a therapist. Because of this, there was no stopping point. People close to her might have recognized that frequent reassuring, interpreting, and predicting would worsen anxiety long-term, or at the very least they would become fatigued. However, ChatGPT was not hindered by such limits. It was always available and agreeable. As a result, reassurance-seeking behaviors could be sustained without interruption, likely reinforcing her underlying anxiety. Even when ChatGPT was initially used to reduce effort rather than to manage uncertainty, anxiety became a perpetuating factor. For example, she might have initially used the tool to summarize a chapter because she was “feeling lazy,” but over time she reported feeling less capable of reading longer texts independently, which in turn likely reinforced her continued use of AI.

Hailey was aware of the sycophantic nature of large language models and attempted to mitigate this by prompting ChatGPT not to “sugarcoat” responses, instructing the tool, “Tell me straight up.” Despite this, she frequently sought reassurance that the responses were not merely agreeable, asking questions such as “Are you just saying that?” At times, ChatGPT would generate responses prefaced with statements such as “It’s going to be hard for you to read this,” which Hailey experienced as increasing credibility and reducing concerns about placation. However, she continued to seek further confirmation that the responses were not designed simply to appease her.

Hailey brought up concerns about her AI use in therapy when she noticed that it was decreasing her confidence. She had written essays in high school and university independently, and this had been a source of confidence. She stated, “I would come home after a night out and write an essay in a few hours—I was good at it. Now [when faced with a writing task], I just stare at the screen and can’t write a sentence.” When asked if she felt this was more an issue of competence vs a problem with confidence, Hailey paused, reflected on the question, and finally concluded that it was more an issue of confidence. Although, as mentioned earlier, she wondered how she had once been able to complete tasks before having access to AI, she could not deny that she had managed successfully until adulthood and could learn to do it again.

## Discussion

### Principal Findings

Hailey’s pattern of AI use can be understood using the I-PACE model [12]. Predisposing factors (person), including mild anxiety and low confidence, contributed to discomfort in situations involving uncertainty. The use of AI provided immediate relief and reassurance (affect), reinforcing repeated engagement. Over time, this was associated with changes in thinking patterns (cognition), including increased reliance on AI for writing, decision-making, and social interpretation, alongside reduced trust in independent judgment. Behaviorally (execution), use became more habitual and, in some contexts, she believed using AI to be necessary for task completion. This progression suggests a shift from initial gratification (efficiency and improved output) toward compensatory use aimed at managing uncertainty and self-doubt.

Hailey’s case demonstrates that even in the absence of significant functional impairment, heavy reliance on generative AI may subtly alter cognitive habits, reduce confidence in independent thinking, and increase dependence on external systems for everyday functioning. Although reassurance seeking appeared to be a central mechanism driving her engagement with ChatGPT, her use extended to offloading judgment. While initially she used it as a tool or an assistant, it later became a source of authority, which made her question her own capacity. Automation bias describes the tendency to trust automated aids over one’s own judgment, which can lead to complacency [13]. Her baseline self-doubt likely made her vulnerable to automation bias.

There was no clear way to differentiate actual decline from perceived decline. Although this patient’s history makes it likely that perceived skill deficit was—at minimum—exaggerated, there is evidence for the consequences of cognitive offloading and automated decision aids [13-16], as well as evidence that the use of AI leads to deskilling [1,17]. As AI tools become more capable and accessible, further research will be needed to understand their long-term cognitive and psychological effects—not only in clinical populations but also in otherwise healthy, high-achieving individuals. Hailey was able to challenge doubts about her own ability by reminding herself of what she had achieved prior to discovering AI. Having this frame of reference was likely what allowed insight into her problematic use. It is reasonable to hypothesize that introducing ChatGPT earlier would have had more a problematic impact—perhaps to both her competence and her confidence. Interpretation and decision-making are important developmental tasks in emerging adulthood, associated with reflective awareness (ie, Siegel’s 2013 concept of mindsight [18]), and repeated reliance on generative AI for these processes may limit opportunities for their consolidation.

Hailey described using ChatGPT as a substitute for speaking with a therapist. This is consistent with previous research indicating that young adult women are especially likely to use generative AI for tasks related to mental health and well-being [19], with 1 study suggesting a preference for generative AI over mental health professionals [20]. However, psychotherapy aims to promote independence and autonomy, whereas generative AI models do not foster these therapeutic goals [10,11,21]. Given the increasing ubiquity of generative AI tools, mental health clinicians working with anxious, reassurance-seeking young adults may encounter similar problematic behaviors. The pattern described occurred in an otherwise high-functioning individual without significant baseline functional impairment, suggesting that similar behaviors may not present as an overt pathology. Instead, they may manifest as subtle but reinforcing patterns of reassurance seeking, cognitive offloading, and externalization of judgment that are easily overlooked in standard clinical interviews. This case highlights the potential importance of assessing AI use when evaluating young adults with anxiety.

### Limitations and Future Research

This report has several limitations. The observations were derived from routine clinical care, and standardized measures were not used to assess the progressive reliance on generative AI. The behavioral pattern described in this report relied on clinical observation and patient self-reporting. It cannot confirm a causal relationship between generative AI use and changes in confidence, cognition, or functioning. At this time, there is also no widely accepted validated instrument designed to assess progressive reliance on generative AI in the manner described here. This report provides a hypothesis-based clinical observation. In the future, the I-PACE model could be empirically tested by having patients track generative AI use and record their thoughts and feelings associated with its use. In addition, further research is needed to create a validated measure of reliance on generative AI.

### Conclusions

This case illustrates a pattern of problematic reliance on ChatGPT that can be conceptualized using the I-PACE model. It highlights a reinforcing pattern that might emerge when there is preexisting difficulty tolerating uncertainty and generative AI therefore becomes an authority rather than an assistant. Mental health clinicians may benefit from inquiring about the use of generative AI in anxious young adults.

## Abbreviations

I-PACE: Interaction of Person-Affect-Cognition-Execution
